# Prevalence of Multidrug-Resistant *Pseudomonas aeruginosa* Isolated from Dairy Cattle, Milk, Environment, and Workers’ Hands

**DOI:** 10.3390/microorganisms11112775

**Published:** 2023-11-15

**Authors:** Basma Badawy, Samar Moustafa, Radwa Shata, Mohamed Z. Sayed-Ahmed, Saad S. Alqahtani, Md Sajid Ali, Nawazish Alam, Sarfaraz Ahmad, Nahed Kasem, Elzahara Elbaz, Hesham S. El-Bahkiry, Reda M. Radwan, Adel El-Gohary, Mona M. Elsayed

**Affiliations:** 1 Department of Hygiene and Zoonoses, Faculty of Veterinary Medicine, Mansoura University, Mansoura 35516, Egypt; adelelgohary@yahoo.com (A.E.-G.); dr.monamohy@yahoo.com (M.M.E.); 2 Department of Zoonoses, Faculty of Veterinary Medicine, Benha University, Benha 13518, Egypt; dr_samarmohamed@yahoo.com; 3 Department of Food Hygiene and Control, Faculty of Veterinary Medicine, Mansoura University, Mansoura 35516, Egypt; radwa.reda@yahoo.com (R.S.); nahedkasem92@gmail.com (N.K.); 4 Department of Clinical Pharmacy, College of Pharmacy, Jazan University, Jazan 82722, Saudi Arabia; nrahman@jazanu.edu.sa (N.A.); sriyazahmad@jazanu.edu.sa (S.A.); 5 Department of Clinical Pharmacy, College of Pharmacy, King Khalid University, Abha 62529, Saudi Arabia; ss.alqahtani@kku.edu.sa; 6 Department of Pharmaceutics, College of Pharmacy, Jazan University, Jazan 82722, Saudi Arabia; msali@jazanu.edu.sa; 7 Department of Internal Medicine and Infectious Diseases, Faculty of Veterinary Medicine, Mansoura University, Mansoura 35516, Egypt; dr.alzhraa.6712@gmail.com; 8 Department of Diagnostic Radiography Technology, Faculty of Applied Medical Sciences, Jazan University, Jazan 82722, Saudi Arabia; hbahkiry@jazanu.edu.sa (H.S.E.-B.); rradwan@jazanu.edu.sa (R.M.R.)

**Keywords:** *P. aeruginosa*, dairy cattle, antimicrobial resistance

## Abstract

*Pseudomonas aeruginosa* is an opportunistic pathogen causing severe infection in animals and humans. This study aimed to determine the ecological distribution and prevalence of multidrug-resistant (MDR) *P. aeruginosa* isolated from dairy cattle, the environment, and workers’ hand swabs. Samples (*n* = 440) were collected from farms and households (*n* = 3, each). Rectal swabs, udder skin swabs, milk, workers’ hand swabs, feed, water, water sources, and beddings were collected. Samples were subjected to the bacterial identification of *P. aeruginosa* via 16S rRNA. Antimicrobial resistance (AMR) was detected either phenotypically using an antibiotic susceptibility test or genotypically with AMR resistance genes (ARGs) such as *drf*A, *sul*1, and *erm*B. *P. aeruginosa* was detected on dairy farms and households (10.3–57.5%, respectively), with an average of 23.2%. The resistance of dairy farm strains was observed against sulfamethoxazole, imipenem, cefepime, piperacillin–tazobactam, and gentamycin (100%, 72.7%, 72.7%, 68.8%, and 63.3%, respectively). Meanwhile, the resistance of household strains was observed against sulfamethoxazole, imipenem, amoxicillin, gentamicin, cefepime, and erythromycin by 91.3%, 82.6%, 75.4%, 75.4%, 68.1%, and 63.8%, respectively. The susceptibility of farm strains was detected against norfloxacin, ciprofloxacin, and levofloxacin (90.9%, 84.8%, and 72.7%, respectively). Meanwhile, the susceptibility of household strains was detected against ciprofloxacin, amikacin, and norfloxacin (100%, 84.1%, and 72.5%, respectively). About 81.4% of *P. aeruginosa* strains were MDR. ARGs (*drf*A, *sul*1, and *erm*B) were detected in farm strains (48.5%, 72.7%, and 24.4%, respectively) and household strains (50.7%, 72.5%, and 47.8%, respectively). Almost all *P. aeruginosa* had MAR over 0.2, indicating repeated application of antibiotics. *P. aeruginosa* prevalence was fivefold higher in households than on farms. MDR strains were higher amongst household strains than farm strains.

## 1. Introduction

*Pseudomonas* is considered one of the most universally pervasive bacterial genera in the world. It is found extensively in the external environment of humans and animals. The genus has a diverse habitat, with approximately 200 species and a complex phylogeny, because of its metabolic capacity and broad potential for adaptation to diverse conditions. *Pseudomonas aeruginosa* is a psychotropic foodborne pathogen with high metabolic adaptability and growth capabilities at very low temperatures, supporting its prominent prevalence in several parts of the food chain [1,2]. *P. aeruginosa* is recognised as an infectious agent transmitted via food and water [3]. It is a Gram-negative bacterium with a zoonotic nature that can cause diseases transmitted from animals to humans and vice versa [4]. It is considered an important cause of acute and chronic diseases in humans [5] and animals, including birds or mammals, that can act as reservoirs of bacterial pathogens. Moreover, environmental contamination via *P. aeruginosa* is prevalent [6].

The pathogen has been recognised in clinical and subclinical mastitis cases in dairy animals. As an environmental pathogen, it widely exists in humid areas, such as muddy bedding, dung, contaminated water, wash hoses in milking parlours, spray nozzles, the inner side of bulk tanks, cow teats, and surfaces that may be associated with the contamination of raw milk [5,7]. Thus, the identification of probable contamination sources and the implementation of hygienic measures during the milking process have become a requirement in the growing dairy industry [7]. In most dairy herds, the occurrence of *Pseudomonas* mastitis is sporadic but may cause a serious herd problem occasionally. *Pseudomonas* is usually regarded as an opportunist; that is, it causes disease under a certain condition of debilitation or is secondary to other infectious agents [2]. Numerous risk factors contribute to *Pseudomonas* infection in farm animals. These factors include the use of common or contaminated teat cannulas for intramammary antibiotic infusion, which is involved in the introduction and spread of *Pseudomonas* mastitis; biofilm formation in milking parlours and poor environmental hygiene, which may facilitate intramammary infection [5]; and access to common sources, such as stagnant water ponds, which may account for certain herd health issues, including *Pseudomonas* mastitis [2].

A number of virulence factors are responsible for the pathogenicity of *P. aeruginosa*, namely exotoxins, proteases, elastases, and phenazine pigments, several of which are under the control of a cell-density recognition mechanism called quorum sensing [6]. The bacterium is naturally resistant to numerous antimicrobial agents as a result of its outer membrane-permeability barrier. *Pseudomonas* also maintains antimicrobial resistance (AMR) plasmids, R-factors, and resistance transfer fragments, and it can transfer these genes with a horizontal gene transfer, mostly via transduction and conjugation [2]. The treatment of *P. aeruginosa* infections is of great concern, owing to the organism’s capacity to resist numerous existing and regularly used antimicrobials. The excessive use of antibiotics during treatment also increases the development of multidrug-resistant (MDR) strains, which renders antibiotic therapy ineffective against this microorganism. MDR–*P. aeruginosa* produces antibiotic-resistant genes against β-lactam, fosfomycin, fluoroquinolones, phenicol, sulphonamides, and aminoglycoside antibiotics [8]. Moreover, *P. aeruginosa* has a prominent ability to form biofilms, making it more resistant to antimicrobial action [9].

Complex universal public hazards emerge, owing to AMR, thereby necessitating the application of new antimicrobial agents for therapeutic purposes to combat pathogenic microorganisms. However, no regulation for antibiotic use is implemented in Egypt, and antimicrobials are still applied as growth promoters and feed additives in animal feedstuffs to treat and prevent zoonotic diseases [10]. The present study aimed to detect the ecological distribution and prevalence of MDR–*P. aeruginosa* from dairy cattle, milk, the environment, and workers’ hand swabs.

## 2. Materials and Methods

### 2.1. Study Design

A cross-sectional study was conducted in two different housing sectors for dairy cattle. Random sampling was adopted in the examined sectors. A structured questionnaire was designed and distributed to farm owners to gain general information about each examined animal production sector and to determine the hygiene level in these houses. This study primarily aimed to detect the ecological distribution and prevalence of MDR–*P. aeruginosa* from dairy cattle reared in either organised farms or households, milk, the environment, or workers’ hand swabs.

This work was conducted in accordance with the Declaration of Mansoura University, Egypt. Approval was granted by the Ethics Committee of the Faculty of Veterinary Medicine, Mansoura, Egypt (Ref. code No.: R/144-2022).

### 2.2. Origins and Processing of Samples

A total of 440 samples were collected from two different housing sectors of dairy cattle, specifically well-organised farms or households, in Dakahlia Governorate, Egypt. Amongst the samples, 320 were from three dairy cattle farms, and 120 were from three dairy cattle households. The samples were randomly selected. Samples from the dairy farm comprised rectal swabs, udder skin swabs, milk, feed, water from drinking troughs, and water sources used to wash the udder and milking utensils and bedding materials. Human samples were obtained randomly from workers’ hand swabs. The examined farms were selected on the basis of the owners’ agreements. All the examined dairy cattle houses depended on underground water.

#### 2.2.1. Animal Samples

Milk (10 mL) was collected from each. The quarter samples were pooled per cattle as one sample in a sterile tube after cleaning and disinfecting the udders in ethyl alcohol and after discarding the first stream of the foremilk. For the rectal and udder skin swabs, moistened swabs were gently rotated either inside the rectum or over the udder skin. Ten swabs per cattle were pooled as one sample in a sterile tube.

#### 2.2.2. Environmental Samples

Bedding (100 g) was collected from five different locations in each cattle farm or household and placed as one sample onto a sterile plastic bag. For water samples, 1 L of water was filtered through 0.45 μm sterile nitrocellulose filters (Millipore, Billerica, MA, USA). The filters were then vortexed in tryptone soya broth, and bacteria were allowed to grow. The water samples were collected either from water troughs in front of cattle or from the water source used in cattle drinking, washing of the udder, and washing of milking utensils. Feed samples (weighing around 100 g per sample) were collected from feed troughs in front of cattle and placed into a sterile plastic bag.

#### 2.2.3. Human Samples

The moistened swabs were gently rotated over the hands of workers. Ten swabs per worker were pooled as one sample onto a sterile tube.

### 2.3. Animal-House Description

Dairy farms I (accommodating 704 dairy cattle) and II (accommodating 398 dairy cattle) had a history of a sharp decrease in milk production with a moderate level of adoption of hygienic measures. Farm III (accommodating 545 healthy dairy cattle) had no history of milk production drops. Good hygienic measures were adopted either in the rest area, feed storage area, or milking parlour, and the milking order was implemented properly. All dairy cattle in examined households (accommodating a few numbers of dairy cattle) had a history of subclinical mastitis. No hygienic measures were adopted for the house nor the udder. Upon asking the owners about antibiotics that were used on animals with health issues, the most frequently used antibiotics in farms and households were sulphonamides, trimethoprims, and macrolides.

### 2.4. Bacteriological and Chemical Identification of P. aeruginosa Strains

Dairy cattle samples, their environment, and human hand swabs were obtained aseptically and inoculated in tryptone soya broth (Oxoid, UK) before agar plating, followed by a *Pseudomonas* agar base (Oxoid, Basingstoke, UK) with a cetrimide nalidixic acid supplement, in accordance with the method described by Ibrahim [11]. Then, the plates were aerobically incubated at 37 °C and examined after 24–48 h. Suspected colonies (3–5) characterised by pigmented colonies (either brown or green) with a musty smell were collected and subjected to further biochemical identifications. Isolation and chemical identification were conducted in the Hygiene and Zoonoses Laboratory, Faculty of Veterinary Medicine, Mansoura University, Egypt. Only one biochemically identified isolate from each pooled sample was subjected to further molecular identification and resistance checking for easy calculation of *P. aeruginosa* prevalence isolated from different samples. Finally, the suspected isolates were sent to the Animal Health Research Institute (Dokki, Cairo, Egypt) for further molecular characterization.

### 2.5. Molecular Identification of P. aeruginosa via 16S rRNA Gene Detection

DNA was extracted from all biochemically identified *P. aeruginosa* isolates (n = 102) incubated overnight in TSB broth using a QIAamp DNA Mini kit (Qiagen, Hilden, Germany, GmbH) in accordance with the manufacturer’s recommendations. In a typical procedure, a 200 µL sample suspension was incubated with 10 µL of proteinase K and 200 µL of lysis buffer at 56 °C for 10 min. After incubation, 200 µL of 100% ethanol was added to the lysate. The sample was then washed and centrifuged following the manufacturer’s recommendations. Nucleic acid was eluted with the 100 µL elution buffer provided in the kit. The primers were supplied by Metabion (Planegg, Germany) and used under certain conditions (Table 1). The primers were utilised in a 25 µL reaction containing 12.5 µL of Emerald Amp Max polymerase chain reaction (PCR) Master Mix (Takara, Osaka, Japan), 1 µL of each primer at a 20 pmol concentration, 5.5 µL of water, and 5 µL of DNA template. The reaction was performed in an Applied Biosystems 2720 Thermal Cycler (Thermo Fisher Scientific, Waltham, MA, USA). The PCR products were separated via electrophoresis on 1% agarose gel (Applichem, Darmstadt, Germany, GmbH) in a 1× TBE buffer at room temperature with 5 V/cm gradients. For gel analysis, 40 µL of products was loaded into each gel slot. A gene ruler with a 100 bp ladder (Fermentas, Thermo, Kandel, Germany) was used to determine the fragment sizes. The gel was photographed with a gel-documentation system (Alpha Innotech, Biometra, San Leandro, CA, USA), and the data were analysed using computer software.

### 2.6. Phenotypic AMR of Farms and Households’ Strains of P. aeruginosa

Antimicrobial susceptibility tests (ASTs) were performed using the agar disc diffusion method on Mueller–Hinton agar (Difco, Franklin Lakes, NJ, USA) as recommended by the Clinical and Laboratory Standards Institute (CLSI). Frequently applied antibiotics for humans and animals were selected to be tested against our isolated strains of *P. aeruginosa.* Eleven antimicrobial discs (Oxoid, Basingstoke, Hampshire, UK) related to different classes of antibiotics were used. They were the macrolide erythromycin (E, 15 µg); the aminoglycosides gentamicin (G, 30 µg) and amikacin (AK, 30 µg); the folate pathway inhibitor trimethoprim–sulphamethoxazole (SXT, 25 µg); the fluoroquinolones norfloxacin (NOR, 10 µg), ciprofloxacin (CP, 5 µg), and levofloxacin (LEV, 5 µg); the carbapenem imipenem (IPM, 10 µg); the β-lactam amoxicillin (AX, 10 µg); and cefepime (CPM, 30 µg), and the penicillin combined with β-lactamase inhibitors piperacillin–tazobactam (TZP, 110 µg). The examined strains were assessed as susceptible or resistant in accordance with the CLSI guidelines for *P. aeruginosa* ATCC^®^a 27853 [16,17]. ASTs were performed in triplicate. To ensure data compatibility, we repeated the experiment with positive and negative controls. The positive control (quality-control organism) was *P. aeruginosa* ATCC^®^a 27853. The negative control was 30 µL of sterile distilled water pipetted onto a blank disc (diameter = 6 mm). AMR data were accessible only when the quality-control test findings were within acceptable ranges. The multiple AMR (MAR) index was calculated by dividing the total number of AMRs for each isolate by the total number of tested antimicrobial agents [10,18]. MDR–*P. aeruginosa* was defined as *P. aeruginosa* not susceptible to at least one antibiotic in at least three antibiotic classes for which *P. aeruginosa* susceptibility was generally expected, namely, penicillin, cephalosporins, fluoroquinolones, aminoglycosides, and carbapenems [19].

### 2.7. Molecular Identification of P. aeruginosa AMR-Resistance Genes (ARGs)

Given that the most frequently applied antibiotics in the examined houses were sulphonamides and macrolides, primers for *drf*A, *sul*1, and *erm*B were selected and supplied from Metabion (Germany) under certain conditions (Table 1). Uniplex PCR was conducted on each gene in accordance with previously described methods [13,14,15].

### 2.8. Statistical Analysis

The normality of the data was first tested with a one-sample Kolmogorov–Smirnov test. A Chi-square test was performed to analyse the data for comparing two or more groups of categorical variables using Statistical Analysis Software (SAS, Software version 9.4, SAS Institute, Cary, NC, USA). A comparison was conducted between the different farms examined. A comparison was conducted between the prevalence of bacteria in different animal houses, either from the animals themselves, their environment, or workers’ hand swabs. The most prevalent source of *P. aeruginosa* contamination amongst different animal, environmental, and human samples was detected. The AMR and susceptibility between different sources were further detected at the level of the examined farms and households. The most prevalent antimicrobial genes were identified within the two examined housing sectors and between different sources of examined samples. A comparison was performed between the occurrence, phenotypic, and genotypic AMR of microorganisms amongst different sources, either animal, environmental, or human samples, as well as amongst different sectors of animal housing. The significance level was *p* < 0.05.

## 3. Results

### 3.1. Prevalence of P. aeruginosa Isolated from Three Examined Dairy Farms and Households

The prevalence of *P. aeruginosa* was based on 16S rRNA detection. The prevalence of *P. aeruginosa* in households (57.5%) was fivefold higher than that in farms (10.6%). The prevalence of *P. aeruginosa* was higher in animal samples (26.4%) than environmental samples (25.4%) in all examined houses (Table 2). Human hand swabs showed a low prevalence rate (8.3%). A significant difference (*p* = 0.045) was observed in the prevalence of *P. aeruginosa* isolated from all the examined dairy farms (Table 3), in which the animal samples revealed a higher prevalence than environmental. Milk displayed the lowest prevalence amongst animal samples (10%), but rectal and udder skin swabs exhibited the highest values (20.8% and 15.6%, respectively). Meanwhile, the highest prevalence values amongst environmental samples were observed in bedding materials (10%), drinking water (9.7%), and water sources used for udder washing (8.3%). Workers’ hand swab samples showed the lowest prevalence (2.2%). The prevalence of *P. aeruginosa* was higher in farm II (16.4%) than in farms I (13.3%) and III (5%).

Regarding the examined dairy households, no significant difference was observed amongst the animal, environmental, and worker samples (Table 3). Dairy households showed a higher prevalence of *P. aeruginosa* in animal samples (62.2%) than the environment (61.7%) (*p* = 0.516). Rectal and udder skin swabs showed the highest percentage (66.7% each), followed by milk (53.3%). Feedstuffs, drinking water (73.3% each), and bedding and water sources (53.3% and 46.7%, respectively) showed higher recovery rates amongst environmental samples, with no significant difference between different sample sources (*p* = 0.516). The prevalence of *P. aeruginosa* was higher in household I (71.7%) than in households II (60%) and III (47.5%).

### 3.2. AMR of P. aeruginosa Recovered from Different Sources

According to the results obtained and interpreted from AST, the resistance of dairy farm strains was observed against SXT, IPM, CPM, TZP, and G by 100%, 72.7%, 72.7%, 68.8%, and 63.3%, respectively. Meanwhile, the susceptibility of farm strains was detected against NOR, CP, and LEV (90.9%, 84.8%, and 72.2%, respectively) (Table 4). The resistance of household strains was observed against SXT, IPM, AX, G, CPM, and E by 91.3%, 82.6%, 75.4%, 75.4%, 68.1%, and 63.8%, respectively). Meanwhile, the susceptibility of household strains was detected against CP, amikacin, and NOR (100%, 84.1%, and 72.5%, respectively) (Table 5).

Regarding dairy-farm ARGs, about 16, 24, and 14 strains were found to be positive for *drf*A, *sul*1, and *erm*B, respectively. Seven strains carried *drf*A and *sul*1 simultaneously. The three examined ARGs were concurrently detected in six dairy-farm strains. Out of 33 positive *P. aeruginosa* strains, 26 strains were MDR (78.8%) (Table 6). For household ARGs, about 35, 50, and 33 strains were found to be positive for *drf*A, *sul*1, and *erm*B, respectively. Twenty-four strains carried *drf*A and *sul*1 simultaneously. The three examined ARGs were concurrently detected in 13 household strains. Amongst the 69 positive *P. aeruginosa* strains, 57 strains were MDR (82.6%) (Table 7). Approximately all positive strains from examined farms and households had “a MAR” exceeding 0.2, indicating a high-risk contamination source in which antibiotics were repeatedly applied.

## 4. Discussion

*P. aeruginosa* had a wide-ranging prevalence in the two examined sectors (farms and households). Regarding the prevalence of *P. aeruginosa* recovered from examined dairy cattle farms, the results of research conducted in Malawi [20] coincided with ours. Our research detected *Pseudomonas* species at a rate of 10.2%, which was lower than the values previously reported by other authors (11.7% and 29.6%) [7,21]. The detection rate of *P. aeruginosa* from rectal swabs of dairy cows in this work was lower than a previously reported one in Egypt [21], which detected the organism (34%) from faecal matter from dairy cattle farms. The recovery rate of *P. aeruginosa* from milk was higher than (5.4%) that of milk samples collected from bovine subclinical mastitis animals in Pengal [22] and lower than those recovered from milk and milk tank samples (70% and 24%, respectively) [7]. Conversely, in another study conducted in Malawi, *Pseudomonas* species cannot be isolated from milk samples [20]. This finding may be owing to the failed cleaning process of containers to meet standards; that is, more than 80% of the dairy farmers do not disinfect their milk-handling containers after cleaning [23]. The contamination rate of water samples in our study was nearly similar to other research findings [24,25]. Higher detection rates of water, feedstuffs, and environmental contamination by *P. aeruginosa* have been previously documented [7,20]. In studies conducted in Egypt, the recovery rates of *P. aeruginosa* from workers’ hand swabs (20% and 28%) are higher than those in the present study [7,21]. The isolation of *P. aeruginosa* from rectal swabs from dairy cows indicated the dissemination of organisms between farm animals and their environment, as previously mentioned by Elshafiee et al. [24]. *P. aeruginosa* was detected in water used for udder washing, consistent with the findings of Kirk and Bartlett [26]. This result confirmed the persistent contamination of wash water, wash hoses, and spray nozzles in the parlour, which led to reinfection by *P. aeruginosa* causing clinical mastitis and chronic infections. Correspondingly, the organism was detected in udder skin swabs in the present study, possibly owing to insufficient udder hygiene before, during, and after the milking process. This result was supported by the work of Schauer et al. [5], who isolated the organism from the disinfectant solution and microfiber towels used for teat cleaning. The prevalence of *P. aeruginosa* was fivefold higher in households than farms, possibly owing to the lack of all hygienic measures in this sector of housing without any veterinary supervision.

A 5-year National Action Plan on AMR (2017–2022) was officially launched in numerous countries, including Egypt. Its strategic purposes are correlated with the consistent investigation of AMR and the optimisation of antimicrobial drug management in human medicine and animal health under the One Health concept released by WHO (2017). This concept aims to establish the interconnectivity of animal and human health with each other and their environments [10,18]. AMR findings from dairy cattle samples in the present study agreed with previous ones that have detected 50% gentamycin resistance [25], high resistance to E, SXT [27], high resistance to AX [28], and the susceptibility of *P. aeruginosa* strains to CP [5]. Conversely, CP resistance has been previously documented [25], as well as the high sensitivity towards G and IPM [27] and the full susceptibility of *P. aeruginosa* to IPM [28]. Antibiotic resistance was higher in households’ strains than in farm strains, probably owing to the massive use of antibiotics without veterinary supervision. The high resistance of *P. aeruginosa* strains to SXT was attributed to the frequent application of sulphonamides in the examined houses in the current work. The reason may be the extensive use of sulphonamides as synthetic veterinary antibiotics in numerous countries owing to their low costs [29].

The MAR index in this research ranged from 0.090 to 0.818 with a high MDR (81.4%), reflecting a great public-health hazard caused by difficulties in treating *Pseudomonas* infection in humans and animals. Our observation of ”a MAR” of *P. aeruginosa* strains matched the observation of Mahmoud [25], who recorded “a MAR” index ranging from 0.44 to 0.77 and observed that six and one MDR strains originate from cows and their drinking water samples, respectively. Other researchers have recorded an elevated “a MAR” index from mastitic milk (ranging from 0.5 to 0.8) and 100% MDR in all recovered strains [28], with an MDR index of 0.8 for two strains. This multidrug resistance of recovered strains may result from the uncritical use of antibiotics in daily farm practice. The presence of AMR genes *drf*A, *sul*1, and *erm*B in animals and their environment can be attributed to the frequent usage of sulphonamides, trimethoprims, and macrolides in the examined farm animals. The *drf*A and *sul*1 genes in human samples collected from farm and household workers may be acquired from animals and their environment. The close contact between animal and human populations may be a high-risk factor for developing such a bacterial infection [24]. Sulphonamide resistance encoded by *sul*1 has been previously documented (63.6%) in water samples from different sources and human ear swabs [30]. *Pseudomonas* is one of the most prevalent *sul-*positive genera in soil fertilised using animal manure, suggesting a potential human-health risk [29]. Studies on livestock or their products have recorded the susceptibility of *P. aeruginosa* isolates to SXT, which was attributed to mutations in mex gene determinants and in mutL and mutS [30].

Study limitation: multi-locus sequence typing is needed in future studies to provide more precise and valuable information on the identity amongst different sequences and to illustrate the genetic relatedness amongst the retrieved *P. aeruginosa* strains.

## 5. Conclusions

The household dairy sector (family farms) was contaminated by *P. aeruginosa* five times more than dairy farms. *P. aeruginosa* was found to have AMR against SXT, IPM, AX, G, and CPM. Meanwhile, *P. aeruginosa* was found to have antimicrobial susceptibility against amikacin, CP, and NOR in both examined sectors. The percentage of MDR strains was higher in household strains than in farm strains. All our findings indicated that the household sector, which is commonly present in Egyptian villages, represents a serious route of AMR dissemination.

## Figures and Tables

**Table 1:** Target genes of *P. aeruginosa* with their primer sequences under certain conditions.

Target Gene	Primer Sequence (5′–3′)	Amplified Segment (bp)	Primary Denaturation	Amplification (35 Cycles)			Final Extension	Reference
		
*16S rRNA*	GGGGGATCTTCGGACCTCA TCCTTAGAGTGCCCACCCG	956	94 °C/5 min	94 °C/30 s	58 °C/40 s	72 °C/45 s	72 °C/10 min	[12]
*sul*1	CGGCGTGGGCTACCTGAACG GCCGATCGCGTGAAGTTCCG	433	94 °C/5 min	94 °C/30 s	60 °C/40 s	72 °C/45 s	72 °C/10 min	[13]
*drf*A	TGGTAGCTATATCGAAGAATGGAGT TATGTTAGAGGCGAAGTCTTGGGTA	425	94 °C/5 min	94 °C30 s	60 °C/40 s	72 °C/45 s	72 °C/10 min	[14]
*erm*B	CATTTAACGACGAAACTGGC GGAACATCTGTGGTATGGCG	425	94 °C/5 min	94 °C/30 s	51 °C/40 s	72 °C/45 s	72 °C/10 min	[15]

**Table 2:** Prevalence of *P. aeruginosa* in examined dairy farms (cattle, environment, and human).

Samples	Farm I		Farm II		Farm III		Total of Examined Farms (*n* = 3)	
	Total No.	Positive No. (%)	Total No.	Positive No. (%)	Total No.	Positive No. (%)	Total No.	Positive No. (%)
Animal	60	13 (21.7)	33	6 (18.2)	55	4 (7.3)	148	23 (15.6)
Rectal swabs	20	5 (25)	13	3 (23.1)	20	3 (15)	53	11 (20.8)
Milk	20	3 (15)	10	1 (10)	20	1 (5)	50	5 (10)
Udder skin swabs	20	5 (25)	10	2 (20)	15	0 (0)	45	7 (15.6)
Environment	60	4 (6.7)	22	3 (13.6)	45	2 (4.4)	127	9 (7.1)
Drinking water	15	2 (13.3)	6	1 (16.7)	10	1 (10)	31	3 (9.7)
Water source	15	1 (6.7)	6	1 (16.7)	15	0 (0)	36	3 (8.3)
Feedstuff	15	0 (0)	5	0 (0)	10	0 (0)	30	0 (0)
Bedding	15	1 (6.7)	5	1 (20)	10	1 (10)	30	3 (10)
Human (hand swabs)	15	1 (6.7)	10	0 (0)	20	0 (0)	45	1 (2.2)
Total	135	18 (13.3)	65	9 (16.4)	120	6 (5)	320	33 (10.3)
*p* value		*p* = 0.267		*p* = 0.914		*p* = 0.36		*p* = 0.045 *

* Significant difference (*p* < 0.05), *p* value: difference between sources within each farm.

**Table 3:** Prevalence of *P. aeruginosa* isolated from three examined dairy households (cattle, environment, and human).

Samples	Household I		Household II		Household III		Total of Examined Household (*n* = 3)	
	Total *n*	Positive *n* (%)	Total *n*	Positive *n* (%)	Total *n*	Positive *n* (%)	Total *n*	Positive *n* (%)
Animal	15	11 (73.3)	15	8 (53.3)	15	9 (60)	45	28 (62.2)
Rectal swabs	5	3 (60)	5	5 (100)	5	2 (40)	15	10 (66.7)
Milk	5	4 (80)	5	1 (20)	5	3 (60)	15	8 (53.3)
Udder skin swabs	5	4 (80)	5	2 (40)	5	4 (80)	15	10 (66.7)
Environment	20	14 (70)	20	14 (70)	20	9 (45)	60	37 (61.7)
Drinking water	5	4 (80)	5	5 (100)	5	2 (40)	15	11 (73.3)
Water source	5	3 (60)	5	3 (60)	5	1 (20)	15	7 (46.7)
Feedstuff	5	5 (100)	5	3 (60)	5	3 (60)	15	11 (73.3)
Bedding	5	2 (40)	5	3 (60)	5	3 (60)	15	8 (53.3)
Human (hand swabs)	5	1 (20)	5	2 (40)	5	1 (20)	15	4 (26.7)
Total	35	25 (71.7)	40	24 (60)	40	19 (47.5)	120	69 (57.5)
*p* value		*p* = 0.813		*p* = 0.402		*p* = 0.287		*p* = 0.546 *

* *p* value: difference between sources within each household.

**Table 4:** Distribution of AMR of *P. aeruginosa* farm strains (*n* = 33) based on AST results.

Samples		Total No. of Isolates	Distribution of Antimicrobial Resistance Amongst Strains										
			E	G	AK	CP	NOR	SXT	IMP	AX	CPM	LEV	TZP
Farm I	Animal	13	6	11	6	2	0	13	9	4	10	3	7
	Environment	4	1	3	0	0	0	4	3	2	3	0	4
	Human	1	1	1	1	0	0	1	1	1	1	1	1
	Total	18	8	15	7	2	0	18	13	7	14	4	12
Farm II	Animal	6	5	4	4	1	0	6	5	5	4	1	5
	Environment	3	0	0	0	1	0	3	3	3	2	0	2
	Human	0	0	0	0	0	0	0	0	0	0	0	0
	Total	9	5	4	4	2	0	9	8	8	6	1	7
Farm III	Animal	4	1	2	0	1	3	4	1	1	3	2	2
	Environment	2	0	0	0	0	0	2	2	2	1	2	1
	Human	0	0	0	0	0	0	0	0	0	0	0	0
	Total	6	1	2	0	1	3	6	3	3	4	4	3
Total		33	13	21	11	5	3	33	24	18	24	9	22
Resistance %		100%	39.4	63.3	33.3	15	9.1	100	72.7	54.5	72.7	27.3	68.8

E, erythromycin; G, gentamicin; AK, amikacin; CP, ciprofloxacin; NOR, norfloxacin; SXT, trimethoprim–sulphamethoxazole; IMP, imipenem; AX, amoxicillin; CPM, cefepime; LEV, levofloxacin; and TZP, piperacillin–tazobactam.

**Table 5:** Distribution of AMR of *P. aeruginosa* household strains (*n* = 69) based on AST results.

Samples		Total No. of Positive	Distribution of Antimicrobial Resistance Amongst Strains										
			E	G	AK	CP	NOR	SXT	IMP	AX	CPM	LEV	TZP
Household I	Animal	11	2	9	4	0	2	11	9	9	7	2	8
	Environment	14	3	5	4	0	1	14	12	12	10	4	7
	Human	1	0	0	0	0	0	1	0	0	1	0	0
	Total	26	5	14	8	0	3	26	21	21	18	6	15
household II	Animal	8	7	6	1	0	4	8	7	7	4	5	3
	Environment	14	14	14	0	0	9	14	10	10	9	11	7
	Human	2	2	2	0	0	0	2	2	2	2	0	0
	Total	24	23	22	1	0	13	24	19	19	15	16	10
Household III	Animal	9	9	9	0	0	3	6	8	8	9	5	2
	Environment	9	7	7	2	0	0	6	8	4	5	3	0
	Human	1	0	0	0	0	0	1	1	0	0	0	0
	Total	19	16	16	2	0	3	13	17	12	14	8	2
Total		69	44	52	11	0	19	63	57	52	47	30	27
Resistance %		100	63.8	75.4	15.9	0	27.5	91.3	82.6	75.4	68.1	43.5	39.1

E, erythromycin; G, gentamicin; AK, amikacin; CP, ciprofloxacin; NOR, norfloxacin; SXT, trimethoprim–sulphamethoxazole; IMP, imipenem; AX, amoxicillin; CPM, cefepime; LEV, levofloxacin; and TZP, piperacillin–tazobactam.

**Table 6:** Distribution of phenotypic and genotypic AMR profiles of *P. aeruginosa* farm strains (*n* = 33) to the tested antibiotics (*n* = 11).

Source		Sample		Antimicrobial Profile	MAR Index	Distribution of Antibiotic Resistance Genes		
Farm No.	Type	ID	Type			*drf*A	*sul*1	*erm*B
Farm I	Animal	2	Rectal swab	SXT, E, G, AK, IMP, CPM, TZP	0.636 ^MDR^	+	+	+
		6	Rectal swab	SXT, E, G, IMP, AX, CPM	0.545 ^MDR^	+	+	+
		7	Rectal swab	SXT, E, G, AK, CP, CPM	0.545 ^MDR^	+	+	+
		13	Rectal swab	SXT, G, IMP, AX, CPM	0.454 ^MDR^		+	
		19	Rectal swab	SXT, G, AK, IMP, AX, CPM, LEV	0.636 ^MDR^		+	
		33	Milk	SXT, G, AK, CPM, LEV	0.454 ^MDR^		+	
		35	Milk	SXT, E, G, AK, IMP, AX, CPM, TZP	0.727 ^MDR^			+
		39	Milk	SXT, G, IMP, TZP	0.363 ^MDR^		+	
		44	Rectal swab	SXT, G, AK, CPM, TZP	0.454 ^MDR^		+	
		50	Rectal swab	SXT, G, IMP	0.272		+	
		51	Rectal swab	SXT, IMP, CPM, TZP	0.363 ^MDR^		+	
		53	Rectal swab	SXT, E, IMP, TZP	0.363		+	+
		56	Rectal swab	SXT, E, G, CP, CPM, LEV, TZP	0.636 ^MDR^	+		+
	Environment	66	Drinking water	SXT, G, IMP, CPM, TZP	0.454 ^MDR^		+	+
		67	Drinking water	SXT, G, TZP	0.272		+	+
		73	Drinking water	SXT, E, G, IMP, AX, CPM, TZP	0.636 ^MDR^	+		+
		89	Bedding	SXT, G, IMP, AX, CPM, TZP	0.545 ^MDR^		+	
		101	Hand swab	SXT, E, G, AK, IMP, AX, CPM, LEV, TZP	0.818 ^MDR^	+	+	+
Farm II	Animal	115	Rectal swab	SXT, G, AK, IMP, AX, CPM, TZP	0.636 ^MDR^		+	
		119	Rectal swab	SXT, G, AK, IMP, AX, TZP	0.545 ^MDR^		+	
		120	Rectal swab	SXT, E, AK, IMP, AX, CPM, TZP	0.636 ^MDR^	+	+	+
		139	Milk	SXT, E, G, AK, IMP, CPM, LEV, TZP	0.727 ^MDR^	+	+	+
		161	Udder skin swab	SXT, E, IMP, AX, TZP	0.454		+	+
		163	Udder skin swab	SXT, E, G, CP, AX, CPM	0.545 ^MDR^	+	+	+
	Environment	177	Drinking water	SXT, CP, IMP, AX, CPM, TZP	0.545 ^MDR^	+		
		183	Water source	SXT, IMP, AX	0.272	+		
		185	Bedding	SXT, IMP, AX, CPM, TZP	0.454 ^MDR^		+	
Farm III	Animal	201	Rectal swab	SXT, IMP, AX, LEV, TZP	0.454 ^MDR^	+		
		213	Rectal swab	SXT, E, G, CP, NOR, CPM	0.545 ^MDR^	+	+	
		224	Rectal swab	SXT, CP, NOR, CPM	0.363	+		
		246	Milk	SXT, G, NOR, CPM, LEV, TZP	0.545 ^MDR^	+		
	Environment	287	Drinking water	SXT, IMP, AX, LEV	0.363	+		
		292	Bedding	SXT, IMP, AX, CPM, LEV, TZP	0.545 ^MDR^		+	

+ (indicate detection and presence of the gene). E, erythromycin; G, gentamicin; AK, amikacin; CP, ciprofloxacin; NOR, norfloxacin; SXT, trimethoprim–sulphamethoxazol; IMP, imipenem; AX, amoxicillin; CPM, cefepime; LEV, levofloxacin; and TZP, piperacillin–tazobactam. ^MDR^ means multidrug-resistant strain.

**Table 7:** Distribution of phenotypic and genotypic AMR profiles of *P. aeruginosa* household strains (*n* = 69) to the tested antibiotics (*n* = 11).

Source		Sample		Antimicrobial Resistance Profile	MAR Index	Distribution of Antibiotic Resistance Genes		
Household No.	Type	ID	Type			*drf*A	*sul*1	*erm*B
Household I	Animal	311	Rectal swab	G, SXT, IMP, AX, CPM	0.454 ^MDR^		+	
		312	Rectal swab	G, SXT, IMP, AX, TZP	0.454 ^MDR^	+		
		314	Rectal swab	AK, SXT, IMP, AX, CPM, LEV	0.545 ^MDR^		+	
		316	Milk	G, SXT, IMP, AX, TZP	0.454 ^MDR^	+		
		317	Milk	SXT, IMP, AX, CPM, TZP	0.454 ^MDR^		+	
		318	Milk	G, SXT, IMP, AX, CPM, TZP	0.545 ^MDR^	+		
		319	Milk	G, AK, SXT, IMP, AX, CPM, LEV	0.636 ^MDR^	+		
		322	Udder skin swab	E, G, AK, NOR, SXT, CPM, TZP	0.636 ^MDR^	+	+	+
		323	Udder skin swab	G, SXT, IMP, AX, TZP	0.454 ^MDR^		+	
		324	Udder skin swab	G, SXT, IMP, AX, TZP	0.454 ^MDR^		+	
		325	Udder skin swab	E, G, AK, NOR, SXT, CPM, TZP	0.636 ^MDR^	+	+	+
	Environment	326	Drinking water	G, AK, SXT, CPM, LEV, TZP	0.545 ^MDR^	+	+	
		328	Drinking water	G, SXT, IMP, AX, CPM, LEV, TZP	0.636 ^MDR^		+	
		329	Drinking water	AK, SXT, IMP, AX, CPM, TZP	0.545 ^MDR^	+	+	
		330	Drinking water	SXT, IMP, AX, CPM, LEV	0.454 ^MDR^	+		
		331	Water source	E, G, SXT, IMP, AX, CPM, LEV, TZP	0.727 ^MDR^	+		+
		333	Water source	E, G, AK, SXT, IMP, AX, CPM	0.636 ^MDR^		+	+
		334	Water source	NOR, SXT, IMP, AX, CPM	0.454 ^MDR^	+	+	
		336	Feedstuff	SXT, IMP, AX, CPM, TZP	0.454 ^MDR^		+	
		337	Feedstuff	SXT, IMP, AX, CPM, TZP	0.454 ^MDR^		+	
		338	Feedstuff	SXT, IMP, AX, TZP	0.363		+	
		339	Feedstuff	SXT, IMP, AX	0.272		+	
		340	Feedstuff	SXT, IMP, AX	0.272		+	
		343	Bedding	SXT, IMP, AX	0.272	+		
		345	Bedding	E, G, AK, SXT, CPM	0.454	+	+	+
	Human	349	Hand swab	SXT, CPM	0.181	+	+	
Household II	Animal	351	Rectal swab	SXT, IMP, AX, LEV, TZP	0.454 ^MDR^	+	+	
		352	Rectal swab	E, AK, SXT, IMP, AX, LEV, TZP	0.636 ^MDR^	+	+	
		353	Rectal swab	E, G, SXT, IMP, AX, CPM	0.545 ^MDR^	+		+
		354	Rectal swab	E, G, NOR, SXT, IMP, AX, CPM, LEV	0.727 ^MDR^		+	
		355	Rectal swab	E, G, NOR, SXT, IMP, AX	0.545	+		
		360	Milk	E, G, NOR, SXT, IMP, AX, LEV, TZP	0.727 ^MDR^	+	+	+
		362	Udder skin swab	E, G, SXT, CPM, LEV	0.454 ^MDR^			
		364	Udder skin swab	E, G, NOR, SXT, IMP, AX, CPM	0.636 ^MDR^		+	+
	Environment	366	Drinking water	E, G, NOR, SXT, IMP, AX, CPM, LEV	0.727 ^MDR^	+	+	+
		367	Drinking water	E, G, SXT, IMP, AX, CPM, LEV, TZP	0.727 ^MDR^	+	+	+
		368	Drinking water	E, G, SXT, IMP, AX, LEV, TZP	0.636 ^MDR^		+	
		369	Drinking water	E, G, NOR, SXT, IMP, AX, CPM, LEV	0.727 ^MDR^		+	
		370	Drinking water	E, G, SXT, CPM, LEV, TZP	0.545 ^MDR^	+	+	+
		371	Water source	E, G, NOR, SXT, IMP, AX, CPM, LEV	0.727 ^MDR^		+	+
		372	Water source	E, G, NOR, SXT, CPM, LEV, TZP	0.636 ^MDR^			
		374	Water source	E, G, NOR, SXT	0.363	+	+	+
		378	Feedstuff	E, G, SXT, CPM, LEV	0.454 ^MDR^		+	+
		379	Feedstuff	E, G, NOR, SXT, IMP, AX	0.545	+	+	
		380	Feedstuff	E, G, SXT, IMP, AX, CPM, LEV, TZP	0.727 ^MDR^		+	
		381	Bedding	E, G, NOR, SXT, IMP, AX, CPM, TZP	0.727 ^MDR^		+	
		384	Bedding	E, G, NOR, SXT, IMP, AX, LEV, TZP	0.727 ^MDR^		+	+
		385	Bedding	E, G, NOR, SXT, IMP, AX, LEV	0.636 ^MDR^		+	+
	Human	386	Hand swab	E, G, SXT, IMP, AX, CPM	0.545 ^MDR^	+	+	+
		388	Hand swab	E, G, SXT, IMP, AX, CPM	0.545 ^MDR^	+	+	+
household III	Animal	393	Rectal swab	E, G, IMP, AX, SXT, CPM	0.545 ^MDR^	+	+	+
		395	Rectal swab	E, G, IMP, AX, SXT, CPM	0.545 ^MDR^	+		+
		398	Milk	E, G, IMP, AX, SXT, CPM, LEV, TZP	0.727 ^MDR^		+	+
		399	Milk	E, G, IMP, AX, SXT, CPM, LEV	0.636 ^MDR^	+	+	+
		400	Milk	E, G, IMP, AX, SXT, CPM	0.545 ^MDR^		+	+
		402	Udder skin swab	E, G, NOR, IMP, AX, SXT, CPM, LEV, TZP	0.818 ^MDR^		+	+
		403	Udder skin swab	E, G, NOR, IMP, AX, CPM, LEV	0.636 ^MDR^			+
		404	Udder skin swab	E, G, NOR, IMP, AX, CPM	0.545 ^MDR^			+
		405	Udder skin swab	E, G, CPM, LEV	0.363 ^MDR^	+		
	Environment	406	Drinking water	E, G, IMP, AX, CPM	0.454 ^MDR^			+
		410	Drinking water	E, G, IMP, AX, CPM, LEV	0.545 ^MDR^			+
		415	Water source	E, G, IMP, AX, CPM	0.454 ^MDR^			+
		418	Feedstuff	E, AK, SXT, IMP, LEV	0.454 ^MDR^	+	+	+
		419	Feedstuff	E, G, SXT, IMP, CPM	0.454 ^MDR^		+	+
		420	Feedstuff	G, SXT, IMP	0272	+		
		421	Bedding	SXT	0.090	+	+	
		423	Bedding	E, G, IMP, AX, LEV	0.454 ^MDR^			+
		425	Bedding	E, G, AK, SXT, CPM	0.454 ^MDR^	+	+	+
	Human	427	Hand swab	SXT, IMP	0.181	+	+	

+ (indicate detection and presence of the gene). E, erythromycin; G, gentamicin; AK, amikacin; CP, ciprofloxacin; NOR, norfloxacin; SXT, trimethoprim–sulphamethoxazole; IMP, imipenem; AX, amoxicillin; CPM, cefepime; LEV, levofloxacin; and TZP, piperacillin–tazobactam. ^MDR^ means multidrug-resistant strain.

## Data Availability

The data supporting the findings of this study are available within the article.
